# Risk adapted transmission prophylaxis to prevent vertical HIV–1 transmission: Effectiveness and safety of an abbreviated regimen of postnatal oral Zidovudine

**DOI:** 10.1186/1471-2393-13-22

**Published:** 2013-01-24

**Authors:** Jennifer Neubert, Maren Pfeffer, Arndt Borkhardt, Tim Niehues, Ortwin Adams, Mareike Bolten, Stefan Reuter, Hans Stannigel, Hans-Juergen Laws

**Affiliations:** 1 Department of Pediatric Oncology, Hematology and Clinical Immunology, Center for Child and Adolescent Health, medical faculty, Heinrich-Heine-University Düsseldorf, Moorenstrasse 5, 40225, Düsseldorf, Germany; 2 Department of Pediatrics, Helios Clinic Krefeld, Lutherplatz 40, 47805, Krefeld, Germany; 3 Institut of Virology, Heinrich-Heine-University Düsseldorf, Düsseldorf, Germany; 4 Department of Obstetrics and Gynaecology, Heinrich-Heine-University Düsseldorf, Düsseldorf, Germany; 5 Department of Gastroenterology, Hepatology and Infectious Diseases, Heinrich-Heine-University Düsseldorf, Düsseldorf, Germany

**Keywords:** HIV, Vertical transmission, Prophylaxis, ZDV

## Abstract

**Background:**

Antiretroviral drugs including zidovudine (ZDV) are effective in reducing HIV mother to child transmission (MTCT), however safety concern remains. The optimal duration of postnatal ZDV has not been established in clinical studies and there is a lack of consensus regarding optimal management. The objective of this study was to investigate the effectiveness and safety of a risk adapted two week course of oral postnatal ZDV as part of a combined intervention to reduce MTCT.

**Methods:**

118 mother infant pairs were treated according to the German-Austrian recommendations for HIV therapy in pregnancy and in HIV exposed newborns between 2000–2010. In the absence of factors associated with an increased HIV–1 transmission risk, children were assigned to the low risk group and treated with an abbreviated postnatal regimen with oral ZDV for 2 weeks. In the presence of risk factors, postnatal ZDV was escalated accordingly.

**Results:**

Of 118 mother-infant pairs 79 were stratified to the low risk group, 27 to the high risk group and 11 to the very high risk group for HIV–1 MTCT. 4 children were lost to follow up. Overall Transmission risk in the group regardless of risk factors and completion of prophylaxis was 1.8% (95% confidence interval (CI) 0.09–6.6). If transmission prophylaxis was complete, transmission risk was 0.9% (95% CI 0.01-5.7). In the low risk group receiving two week oral ZDV transmission risk was 1.4% (95% CI 0.01–8.4)

**Conclusion:**

These data demonstrate the effectiveness of a short neonatal ZDV regimen in infants of women on stable ART and effective HIV–1 suppression. Further evaluation is needed in larger studies.

## Background

One of the major achievements of HIV research was the demonstration in the PACTG 076 trial that the administration of zidovudine (ZDV) given orally to the pregnant woman at 14 to 34 weeks gestation, given intravenously to the mother during labour and given orally to the infant for 6 weeks in the absence of breastfeeding reduced the risk of perinatal transmission by 70% [1]. It is not known whether all three arms of this regimen contributed equally to the reduction in risk. With the further introduction of effective antiretroviral therapy (ART) and scheduled caesarean section transmission rates of 1–2% were reported in resource rich countries [2,3]. With increasing progression in the treatment of HIV disease especially with an increasing proportion of HIV infected women receiving combination ART throughout pregnancy, modifications in the interventions to reduce HIV-1 MTCT have occurred e.g. a more tolerant approach to vaginal delivery for women who achieve significant viral suppression is now recommended in most guidelines [4]. The optimal duration of neonatal zidovudine chemoprophylaxis has not been established in clinical trials and different strategies are recommended in different guidelines. An increasing proportion of infants receiving no neonatal antiretroviral prophylaxis at all has been reported in western Europe, particularly among those whose mothers received antenatal combined ART [5]. Potential short term adverse effects of antiretroviral prophylaxis in infants include haematologic abnormalities and organ dysfunction [6,7]. Furthermore Nucleoside analogue drugs including ZDV have been associated with mitochondrial toxicity in HIV exposed children however evidence remains equivocal [8,9].

Due to safety concerns, the German-Austrian recommendations for HIV–1 therapy in pregnancy and in HIV exposed children have for many years recommended a risk adapted neonatal zidovudine prophylaxis [10-12]. A de-escalation of the six week postnatal component according to the PACTG 076 protocol is recommended for children at low risk of HIV transmission (complete antepartum and intrapartum prophylaxis, low viral load at birth, absence of birth complications) and these children are treated for 2–4 weeks with oral ZDV. This is in contrast with the US guidelines which recommend a 6 week ZDV chemoprophylaxis for all HIV-exposed neonates. A 4 week neonatal chemoprophylaxis is recommended in the united Kingdom for children at low risk for MTCT [13]. For over 10 years we have implemented the German-Austrian recommendations at our centre. ZDV was given orally for two weeks to almost all the neonates stratified to the low risk group. This strategy was started 10 years ago due to safety concerns with the idea of reducing exposure to ZDV and reducing toxicity. We retrospectively evaluated the effectiveness and safety of this risk adapted postnatal transmission prophylaxis regimen. To our knowledge there are no data so far on the effectiveness and safety of a two week postnatal oral ZDV regimen as part of a combined intervention to prevent MTCT.

## Methods

The clinical data of all HIV–1 exposed children born at our centre between January 2000 and December 2010 were retrospectively analysed in our study. Neonates had been stratified to three risk groups according to the German-Austrian recommendations (low risk, high risk, very high risk group). Premature labour, amnionitis, duration of prepartal maternal ART <  4 weeks, lacking prepartal prophylaxis, viral load  >  3,000 copies/ml, incision injury to child, oral intake of bloody amniotic fluid into gastrointestinal or respiratory tract of the newborn were considered as factors associated with increased vertical HIV–1 transmission risk. In the absence of these transmission risk factors children were considered at low risk for transmission and ZDV was given orally for 2 weeks (2 mg/kg every 6 hours). In the high risk group (e.g. premature labour, duration of maternal ART <  4 weeks ) ZDV was given orally for 6 weeks. In the very high risk group (incomplete prophylaxis e.g. lacking prepartal prophylaxis, Amnionitis, viral load  >  10,000 copies/ml), ZDV was combined with lamivudine (3TC) (2 mg/kg every 12 hours) for 6 weeks and nevirapine (NVP). If the mother received a single dose of NVP prepartum a further NVP Dose was given to the newborn at an age of 48 h–72 h. If NVP was not given prepartum, two NVP Doses (each 2 mg/kg) were given postnatally to the newborn (first dose as soon as possible after birth, second dose on the third day of life). When peripartal viral load was missing at the time of delivery neonates were initially stratified to the high risk group. The rationale for the stratification and procedure mentioned above are highlighted in the German-Austrian recommendations [10-12]. This study was approved by the ethics committee of the medical faculty at the Heinrich-Heine-University Düsseldorf (internal study number 3869).

Until 2008 elective caesarean section was the recommended mode of delivery at our centre regardless of the risk group. Since 2009 vaginal delivery is being offered to women with viral load repeatedly below detection limit, in the absence of obstetric complications. These children were stratified to the low risk group but received 4 weeks of oral ZDV. To ensure therapy adherence all HIV exposed neonates were admitted to the hospital and almost all were discharged at the age of 2 weeks. Infants were tested for HIV-1 at the age of 2 weeks, 4–6 weeks, 3–4 months and at the age of 9 months. Day 1 HIV–1 PCR was not done. Loss of maternal antibodies was subsequently confirmed at the age of 15–18 months. Infants were defined as not infected when HIV–1 PCR was negative in at least two separate blood samples, including one sample taken after the age of 3 months.

In view of potential metabolic abnormalities reported with ART, blood tests were done at baseline including a full blood count, biochemical parameters (urea, serum creatinine, amylase, lipase, alanin and aspartate aminotransferase) and these tests were repeated when possible with the HIV diagnostic samples. Due to the different intensity and duration of antiretroviral therapy in the different transmission risk groups, short term safety data in the different groups were compared using the student t-test for unpaired samples (Excel 5.0 software, Microsoft inc.).

### Data analysis

Data were analysed in the groups to which the patients had been assigned. Primary outcome parameter was the efficacy of the risk adapted postnatal transmission prophylaxis regimen (two week oral ZDV in low risk patients). The evaluation of efficacy was based on the percentage of HIV infected infants in each group. Percentages were estimated with their exact 95% confidence intervals (CI). Secondary outcome parameter was the presence of metabolic abnormalities in the different groups. Variables are presented as mean ± SD. For two group comparisons regarding the metabolic abnormalities the student t-test was used. Statistical significance was considered at a probability (p) value ≤ 0,05 on two sided testing. Statistical analysis was performed with Excel 5.0 software (Microsoft inc.). We lacked statistical power to compare the relative effectiveness of an abbreviated oral ZDV regime in low risk patients to other postnatal regimes published elsewhere.

## Results

**Figure 1:**
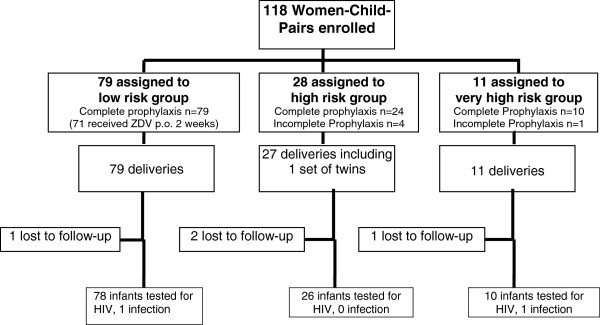
Treatment assignment, loss to follow up, and outcome of pregnancy.

A total of 118 mother-infant pairs including one pair of twins born between January 2000 and December 2010 were included in our study. Treatment assignment, lost to follow up and outcome of pregnancy are shown in Figure 1. 4 children were lost to follow up and were excluded from the final analysis. 71/79 children in the low risk group were treated with oral ZDV for 2 weeks. Base-line characteristics of the mothers including treatment characteristics, viral load, CD4 count, mode of delivery, postnatal prophylaxis and the outcomes of pregnancy are summarised in Table 1.

**Table 1:** Maternal and neonatal characteristics

	**Total**	***n *infected**
	114	2
*Gestational age at delivery*		
< 33. + 0 weeks	1	0
33. + 0-36. + 6 weeks	51	1
≥ 37. + 0 weeks	62	1
*Maternal HIV-1 RNA at delivery*		
< 50	55	0
50–2999	44	1
3000 – 10000	7	0
> 10000	7	1
missing viral load at birth	1	0
*Maternal CD4 cell count at delivery (cells/μl) ( n = 104, missing data n = 10)*		
< 200	11	1
200 – 349	26	1
≥ 350	67	0
*Gender of neonate*		
female	66	2
male	48	0
*Mode of delivery*		
elective cesarean	77	2
emergency cesarean	33	0
vaginal delivery	4	0
*Initiation of antenatal ART (weeks) (n = 112, missing data n = 2)*		
none	3	0
non-stop from onset	24	1
≤ 20	15	0
21 – 28	17	0
> 28	53	1
*Last antenatal art (n = 113, missing data n = 1)*		
HAART	95	2
dual-drug therapy	12	0
Monotherapy	3	0
none	3	0
*Intrapartum prophylaxis*		
Yes	113	2
No	1	0
*Duration of postnatal child prophylaxis (weeks)*		
2	74	2
4	8	0
6	32	0
*Postnatal child prophylaxis*		
dual or HAART	12	0
Monotherapy	102	2
*At-risk-group/risk of infection*		
low risk	78	1
high risk	26	0
very high risk	10	1
*Complete Prophylaxis*		
Yes	105	1
No	9	1

### Analysis of effectiveness (vertical transmission rates)

The rates of HIV–1 transmission were analysed for the entire group including all children even those with incomplete transmission prophylaxis (e.g. missing prepartal prophylaxis, incomplete postnatal prophylaxis) and for the different risk groups separately. In 9 children transmission prophylaxis was incomplete (no antenatal ART n = 3, no intrapartum ZDV n = 1, duration of postnatal prophylaxis too short n = 5). 2 out of 114 infants were infected with HIV in the entire group. Overall Transmission risk in the group including all children even those with incomplete transmission prophylaxis was 1.8% (95% confidence interval (CI) 0.09–6.6). When transmission prophylaxis was completed according to the German-Austrian guidelines (n = 105) overall transmission risk was 0.9% (95% CI 0.01–5.7). Overall transmission risk in the low risk group (n = 70) receiving two week oral ZDV was 1.4% (95% CI 0.01–8.4)

Two children were infected with HIV–1, one child belonging to the very high risk group and one child belonging to the low risk group. In the infected child belonging to the low risk group maternal HIV was first diagnosed during pregnancy, antenatal ART was started with AZT, 3TC and NVP at 30 weeks of gestation, maternal viral load at birth was 449 copies/ml, elective caesarean was done at 36 + 4 weeks of gestation and the child was treated with oral ZDV for 2 weeks. In the infected child belonging to the high risk group HIV–1 Status was known before pregnancy, ongoing ART with 3TC, d4T and NVP was changed to AZT, 3TC and NFV at around 24 weeks of gestation, there were adherence problems during pregnancy, the mother had repeatedly high viral load during pregnancy with HIV-RNA between 11,000 and 32,000 copies/ml, viral load at birth was 11,800 copies/ml. Postnatal chemoprophylaxis was incomplete with only oral ZDV which was discontinued after discharge at the age of 2 weeks and not given for 6 weeks. Both Children were infected before 2005.

### Safety

For safety analysis the low risk group receiving ZDV monotherapy for two weeks (short arm group) was compared with the groups ( high risk group, very high risk group) receiving ZDV > 2 weeks (long arm group). Due to the small patient numbers in the high risk group and very high risk group these two groups were analysed together (long arm group).

### Haematological parameters

We compared the haemoglobin (Hb) concentration, white blood count and platelet count between the short arm group (n = 68) and the long arm group (n = 39) at the age of 2 weeks, 1 month and three months. At the age of 2 weeks the percentage of children with anaemia was 46.5% (Hb 13 ± 3.2 g/dl) in the short arm group and 48.7% (Hb 13.2 ± 3.2 g/dl) in the long arm group. At the age of 1 month anaemia was 34.3% (Hb 10.5 ± 3.4 g/dl) in the short arm group and 60.5% (Hb 10.4 ± 2.9 g/dl) in the long arm however this difference was not significant (t-test, p = 0.612). In general anaemia was mild, however in one child from the high risk group ZDV prophylaxis was discontinued at the age of 5 weeks due to decreased haemoglobin concentration (Hb at the age of 2 weeks 14.4 mg/dl, 1 month 7.8 mg/dl, 3 months 11.9 mg/dl). A significant difference in the white blood count (WBC) in regard to leucopenia was present at the age of 1 month (0% short arm group, 10.5% (4/38) long arm group (short arm group WBC 11,000 ± 4600/μl, long arm group WBC 8600 ±  2800/μl, t-test, p = 4.3 × 10^−6^) however this difference was absent at the age of 3 months with no child in the long arm group having leucopenia at the age of 3 months.

### Biochemical parameters

Biochemical parameters (urea, serum creatinine, amylase, lipase, alanin and aspartate aminotransferase) were compared between the two groups. A significant difference was seen for serum creatinine. Both groups had normal serum creatinine levels (< 0.4 mg/dl) at the age of 2 weeks, however at the age of 1 month 20.6% (7/34) in the long arm group had raised serum creatinine levels (serum creatinine 0.3 ± 0,2 mg/dl) compared with 3.5% (2/57) in the low risk group (serum creatinine 0.3 ± 0,1 mg/dl) (p = 0.05). At the age of 3 months the difference was no longer significant with normal serum creatinine levels in all patients from the long arm group.

## Discussion

Antiretroviral prophylaxis for neonates and for women during pregnancy and delivery has been the cornerstone for prevention of MTCT. Antiretroviral prophylaxis reduces perinatal transmission by several mechanisms, including lowering of maternal prepartum viral load and pre- and post exposure prophylaxis of the infant. Post exposure prophylaxis is provided through administration of ART e.g. ZDV to the infant after birth. This mechanism protects infants from cell free or cell associated virus that might have obtained access to the fetal /infant systemic circulation. In the PACTG 076 trial, ZDV was given orally for 6 weeks and subsequently became standard of care. The benefit of this 6 week postnatal ZDV was demonstrated in the mid–1990s, before combined ART was widely used. It is unclear if a 6 week oral ZDV confers additional benefit for women who achieve viral suppression below detection limit on ART and an increasing proportion of infants receiving no postnatal antiretroviral prophylaxis has been reported in western Europe [5].

Studies and observational data have demonstrated that six weeks of treatment of the infant is beneficial when the mother received little or no treatment [14,15]. In a US cohort study a reduced risk of transmission, compared to no intervention was observed in infants started on ZDV within 48 h of birth [15]. An observational study in Ireland, in which a 4 week course of neonatal oral ZDV was given in combination with maternal antiretroviral prophylaxis, reported a transmission rate of 1% in 964 HIV-exposed infants [16]. Grosch-Wörner et al. demonstrated the effectiveness and safety of a reduced regimen of ZDV prophylaxis including postnatal ZDV given intravenously for 10 days in a small group of patients [17]. In a study performed in Thailand, a short course of only 3 days neonatal ZDV was compared with 6 weeks and there was no increased risk of transmission between the two groups when the mother had received ZDV from 28 weeks of gestation [15]. A review of all currently trialed interventions to prevent MTCT is regularly conducted by the Cochrane HIV/AIDS group and was last published 2011 [18].

In the US Guidelines a 6 week neonatal component of the ZDV chemoprophylaxis is recommended for all HIV-exposed neonates. A 4 week neonatal chemoprophylaxis is recommended in the United Kingdom for children at low risk for MTCT and combined prophylaxis is recommended in special situations e.g. as post delivery prophylaxis when the mother is found to be HIV-infected only after delivery, in unplanned delivery before starting ART or in case of persistent maternal viremia on HAART because of poor adherence or viral rebound due to resistance. Most centres in Germany treat postnatally for 4 weeks with oral ZDV.

In the low risk group receiving oral ZDV for 2 weeks one child was infected with HIV–1. In this child maternal viral load was <  500 copies/ml at the time of birth. Since day 1 HIV–1 PCR was not done, the timing of infection cannot be clarified. The low viral load around birth makes perinatal infection unlikely.

Concerning toxicity and safety, treating neonates with a shorter regimen of ZDV was of short term benefit regarding haematological parameters and kidney function. This benefit may however also be attributed to the combined ART including lamivudine and nevirapine that was given to the patients in the very high risk group.

To our knowledge these are the first data on two week oral ZDV as part of a combined intervention to reduce MTCT of HIV–1. Our data in this small cohort demonstrate that a risk adapted strategy is applicable in a clinical setting and that oral ZDV given for 2 weeks to children at a low risk for MTCT appears to be effective and safe.

## Conclusion

These data support the current trend toward shorter neonatal regimens especially for infants of women who are fully virologically suppressed and on stable ART at the time of delivery. These results need to be confirmed in larger clinical trials and they highlight the need for more risk-benefit analyses to guide clinical practice.

## Competing interests

The authors declare that they have no competing interests.

## Authors’ contributions

JN (pediatrician): conception and design of the study, coordination of the study, acquisition of pediatric data, collection of data, analysis and interpretation of data, drafting of the manuscript. MP (medical student): acquisition of pediatric data, collection of maternal and pediatric data, statistical analysis, revsion of the manuscript, approved the final version of the manuscript. AB (Pediatrician, Head of Department): conception and design of the study, analysis and interpretation of data, critical revision of the manuscript, approved the final version. TN (Pediatrician): conception and design of the study, aquisition of pediatric data, critical revision of the manuscript, approved the final version of the manuscript. OA (Virologist): performed the virologic assays, acquisition of virologic data, critical revision of the manuscript, approved the final version of the manuscript. MB (Gynaecologist): conception and design of the study, acquisition of maternal data, analysis and interpretation of maternal data, critical revision of the manuscript, approved the final version of the manuscript. SR (Internal medicine): conception and design of the study, treatment of the mothers, acquisition of maternal data, analysis and interpretation of maternal data, critical revision of the manuscript, approved the final version. HS (Pediatrician, Neonatologist): acquisition of neonatal data, analysis and interpretation of neonatal data, critical revision of the manuscript, approved the final version. H-JL (Pediatrician): conception and design of the study, acquisition of pediatric data, analysis and interpretation of data, drafting of the manuscript. All authors made a substantial contribution to the study and approved the final version of the manuscript.

## Pre-publication history

The pre-publication history for this paper can be accessed here:

http://www.biomedcentral.com/1471-2393/13/22/prepub
